# Supplementary material for: Impact of study design, contamination, and data characteristics on results and interpretation of microbiome studies
Source: mSystems. 2025 Aug 6;10(9):e00408-25. doi: 10.1128/msystems.00408-25 (PMC12456016; doi:10.1128/msystems.00408-25)

Figure S1. Examples of statistical outcomes for alpha and beta diversity from simulated data, generated by HeritSeq, at the lower, middle, and upper limits of simulated data characteristics. Data characteristics are labelled above graphs in the order of the number of samples in the arbitrary groups (S1,S2), maximum number of taxa in any one sample, and the dissimilarity in microbiome composition between arbitrary groups (higher values reflect greater dissimilarity). p-values are shown on the graph, which reflect Anova (alpha diversity; A-C) or PERMANOVA with 999 permutations (beta diversity; D-F). A and D are simulated with 10 samples per group, a maximum of 10 taxa, and dissimilarity at 0.1. B and E are simulated with 60 samples, 100 taxa, and dissimilarity of 50. C and F are simulated with 240 samples, 5000 taxa, and dissimilarity of 100.

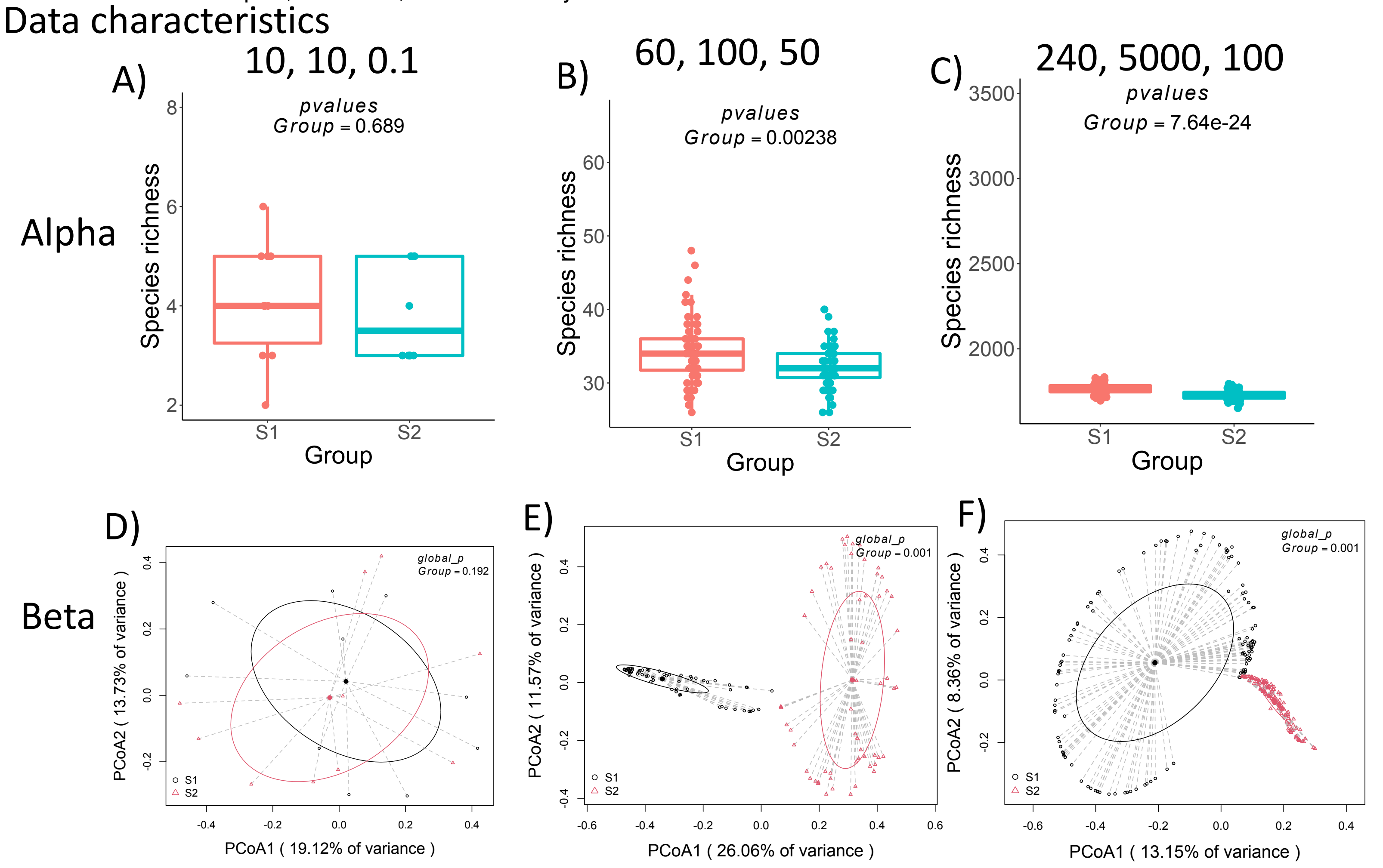

Supplement: Figure S1 — Characteristics of simulated data sets. [file msystems.00408-25-s0004.pdf]
