# Supplementary material for: Impact of study design, contamination, and data characteristics on results and interpretation of microbiome studies
Source: mSystems. 2025 Aug 6;10(9):e00408-25. doi: 10.1128/msystems.00408-25 (PMC12456016; doi:10.1128/msystems.00408-25)

Figure S2. The proportion of significantly different variables in the simulated contaminated datasets that were simulated contaminants, borderline significant in the original dataset (FDR between 0.05-0.1), or random in the original dataset (FDR>0.1). P-values reflect a two-way Anova. The mean % of differentially abundant variables is listed below each boxplot.

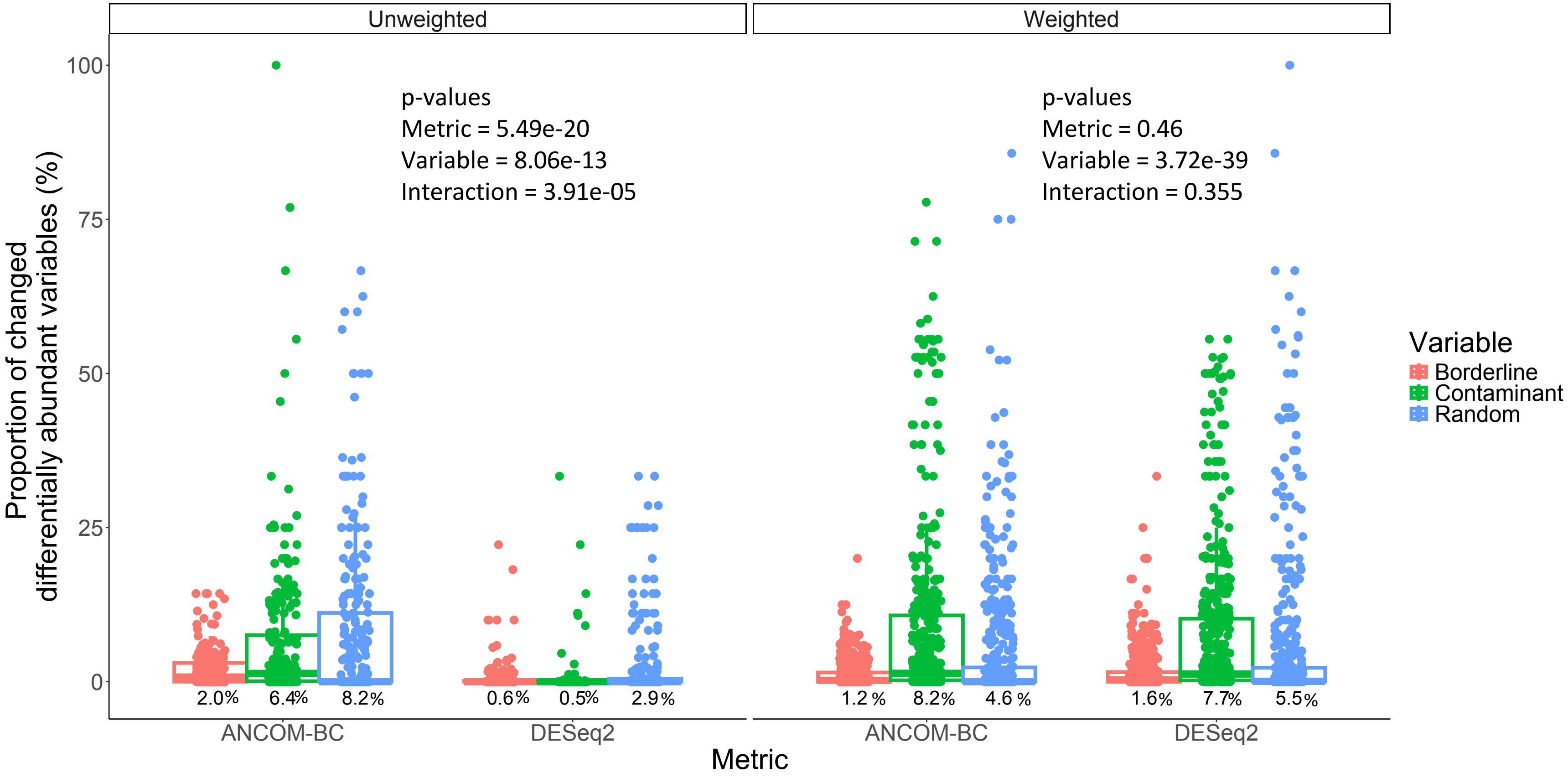

Supplement: Figure S2 — Delineation of false positives due to contamination. [file msystems.00408-25-s0005.pdf]
